# Potential killers exposed: tracking endogenous influenza‐specific CD8^+^ T cells

**DOI:** 10.1111/imcb.12189

**Published:** 2018-08-10

**Authors:** Rachael Keating, Melissa Y Morris, Wen Yue, Cory E Reynolds, Tarsha L Harris, Scott A Brown, Peter C Doherty, Paul G Thomas, Maureen A McGargill

**Affiliations:** ^1^ Department of Immunology St. Jude Children's Research Hospital Memphis TN 38105 USA; ^2^ Department of Microbiology and Immunology University of Melbourne The Peter Doherty Institute for Infection and Immunity Parkville VIC 3010 Australia

**Keywords:** CD8^+^ T‐cell activation, CD8^+^ T‐cell memory, CD8^+^ T cells, influenza infection

## Abstract

Current influenza A virus (IAV) vaccines stimulate antibody responses that are directed against variable regions of the virus, and are therefore ineffective against divergent strains. As CD8^+^ T cells target the highly conserved, internal IAV proteins, they have the potential to increase heterosubtypic immunity. Early T‐cell priming events influence lasting memory, which is required for long‐term protection. However, the early responding, IAV‐specific cells are difficult to monitor because of their low frequencies. Here, we tracked the dissemination of endogenous IAV‐specific CD8^+^ T cells during the initial phases of the immune response following IAV infection. We exposed a significant population of recently activated, CD25^+^CD43^+^ IAV‐specific T cells that were not detected by tetramer staining. By tracking this population, we found that initial T‐cell priming occurred in the mediastinal lymph nodes, which gave rise to the most expansive IAV‐specific CD8^+^ T‐cell population. Subsequently, IAV‐specific CD8^+^ T cells dispersed to the bronchoalveolar lavage and blood, followed by spleen and liver, and finally to the lung. These data provide important insight into the priming and tissue dispersion of an endogenous CD8^+^ T‐cell response. Importantly, the CD25^+^CD43^+^ phenotype identifies an inclusive population of early responding CD8^+^ T cells, which may provide insight into TCR repertoire selection and expansion. A better understanding of this response is critical for designing improved vaccines that target CD8^+^ T cells.

## Introduction

Current influenza A virus (IAV) vaccines are only effective against IAV subtypes included in the vaccine, and therefore do not protect against slightly divergent or novel IAV strains. Thus, developing a successful universal IAV vaccine that induces immunity to regions of IAV that are conserved between different subtypes is of utmost importance. An effective universal vaccine is likely to require both humoral and cellular responses. While the major IAV epitopes targeted by B cells vary between IAV subtypes, CD8^+^ T cells recognize epitopes from internal IAV proteins that are primarily conserved across multiple subtypes.^1^ Thus, vaccines optimizing CD8^+^ T cell‐mediated immunity may enhance protection against novel seasonal and pandemic IAVs.

Mouse models of IAV pneumonia provide a well‐developed experimental system for analyzing CD8^+^ T cell–mediated immunity. In C57BL/6 (H2^b^) mice, the CD8^+^ T‐cell response to the nonlethal H3N2 virus, HKx31, is dominated by CD8^+^ T cells recognizing five viral epitopes: D^b^NP_366_, D^b^PA_224_, K^b^PB1_703_, K^b^NS2_114_, and D^b^PB1‐F2_62_.^2^ Following a primary IAV infection, the majority of the CD8^+^ T‐cell response consists of T cells specific for D^b^NP_366_ and D^b^PA_224_, while the responses directed against the K^b^PB1_703,_ K^b^NS2_11,_ and D^b^PB1‐F2_62_ epitopes are subdominant. However, following secondary infection, D^b^NP_366_‐specific T cells dominate the response, constituting approximately 70% of the IAV‐specific CD8^+^ T cells. Understanding the key determinants that impact CD8^+^ T‐cell activation, expansion, and memory generation during an *in vivo* infection is critical for comprehending these immunodominance patterns and developing improved IAV vaccines.

Following intranasal IAV infection, naïve IAV‐specific CD8^+^ T cells first encounter the viral peptides presented by MHCI complexes on dendritic cells in the mediastinal lymph nodes (MLN), which drain the respiratory tract. TCR diversity ensures that the individual T cells that comprise the responding repertoire bind peptide‐MHCI with variable avidities, with high avidity T cells typically proliferating more extensively following antigen encounter.^3^, ^4^, ^5^ Such recognition of their cognate peptide‐MHCI induces CD8^+^ T‐cell activation, proliferation, and subsequent migration to the lung where effector CTLs directly interact with the infected airway epithelium to lyse infected target cells and limit viral spread. The spleen has also been described as a major priming site for CD8^+^ T cells during IAV infection.^6^ Given that the priming environment impacts differentiation of memory CD8^+^ T cells, it is important to discern the relative contribution of lymph node *versus* splenic priming.

Many factors influence the magnitude of the CD8^+^ T‐cell response following infection. In particular, the cellular environment and the initial priming of naïve CD8^+^ T cells dictate the efficacy of recall responses, and therefore impact vaccine efficacy.^7^, ^8^, ^9^, ^10^ Analysis of the early events of IAV‐specific CD8^+^ T‐cell responses has been limited, in part because numbers of virus‐specific CD8^+^ T cells remain low during the initial stages following antigen encounter. To circumvent this limitation, several groups transferred naïve, TCR transgenic T cells into recipients prior to infection to increase the precursor frequency and hence, responding population.^6^, ^11^ While these studies have provided invaluable insights, their interpretation has been confounded by using unnaturally high CD8^+^ effector T‐cell precursor frequencies.^12^ Furthermore, use of TCR transgenic mice also perturbs the natural diversity in TCR affinity for the peptide‐MHCI complexes, the competition between T cells specific for different viral epitopes, and the timing of antigen exposure and stimulatory microenvironments dictated by antigen presenting cells; all of which impact the immune response.^8^, ^13^, ^14^ Therefore, it is important to study the immune response in an endogenous model represented by naturally occurring TCR specificities and response kinetics.

Magnetic enrichment of antigen‐specific T cells with peptide: MHC tetramers has facilitated isolation of small numbers of endogenous, antigen‐specific CD8^+^ T cells, which has been instrumental in examining the relationship between naïve CD8^+^ T‐cell precursor frequency and the magnitude of the CD8^+^ T‐cell response.^15^, ^16^, ^17^ Initially, CD8^+^ T‐cell frequencies were identified as a strong determinant of the magnitude of the response. However, it is becoming increasingly evident that multiple factors contribute to CD8^+^ T‐cell expansion.^9^, ^18^, ^19^ For example, the number of naïve CD8^+^ T cells specific for D^b^NP_366_ and D^b^PA_224_ is significantly lower than the number of naïve CD8^+^ T cells specific for the K^b^NS2_114_ and D^b^PB1‐F2_62_ epitopes prior to infection. However, as early as 5 days after infection, the D^b^NP_366_ and D^b^PA_224_‐specific T cells significantly outnumber the K^b^NS2_114_ and D^b^PB1‐F2_62_‐specific T cells, indicating that precursory frequency is not the sole determinant of the magnitude of the CD8^+^ T‐cell response.^18^, ^19^ Further experiments demonstrated that the capacity of the T cells to proliferate following IAV infection and the avidity of the TCR for antigen also contribute to the magnitude of the CD8^+^ T‐cell response.

While isolation of T cells with tetramers allows detection of a small number of cells, this approach relies on pooling cells from multiple lymphoid organs, and therefore cannot be used to track the dispersion of small numbers of antigen‐specific T cells to each organ. Furthermore, individual tetramers may only detect a subset of the total pool of antigen‐specific T cells. As the environment in which a T cell encounters antigen influences the capacity of that T cell to become a memory or an effector T cell, we wished to characterize the early priming and tissue dispersion of IAV‐specific CD8^+^ T cells during IAV infection. Additionally, we examined whether specific surface markers could be used to identify IAV‐specific T cells early in the response. We found that the CD25^+^CD43^+^CD8^+^ phenotype provided a more effective measure of IAV‐specific CD8^+^ T‐cell frequencies than tetramer analysis during the early events following priming. By tracking the progression of CD25^+^CD43^+^ CD8^+^ T cells and tetramer‐positive T cells, we showed that priming in the MLN gave rise to the most expansive population of IAV‐specific CD8^+^ T cells. At later time points, the bronchoalveolar lavage (BAL) and lung were enriched for effector CD8^+^ T cells, while the spleen and MLN harbored memory populations. These findings provide insight into the activation and dispersion of endogenous, IAV‐specific CD8^+^ T cells, which is important for developing methods to enhance CD8^+^ T‐cell memory during IAV vaccination.

## Results

### Tissue specific dissemination kinetics of IAV‐specific CD8^+^ T cells

**Figure 1:**
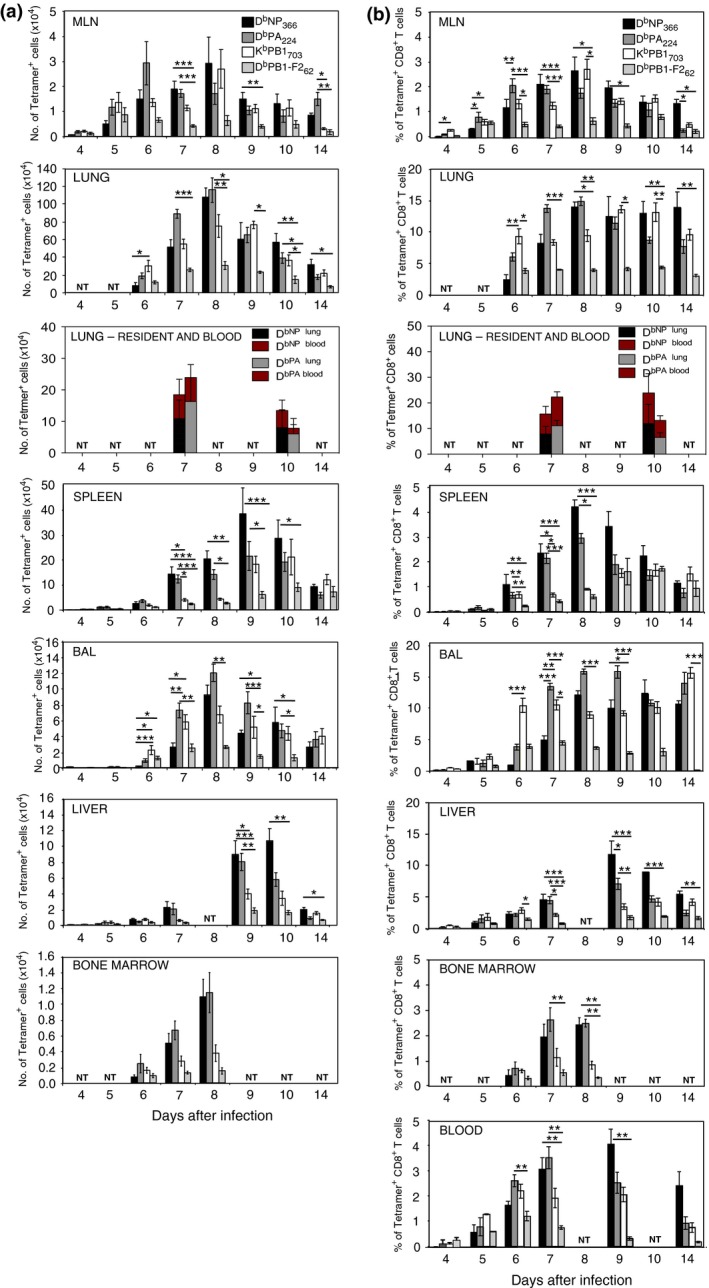
Tissue dispersion of IAV‐specific CD8^+^ T cells during primary IAV infection. Cells were isolated from the indicated tissues following intranasal HKx31 virus infection of naïve mice and were analyzed for D^b^NP_366,_ D^b^PA_366_ , D^b^PB1_366,_ and D^b^PB1‐F2 tetramer binding and CD8α expression. Each bar indicates the mean ± s.e.m. **(a)** number or **(b)** percentage of D^b^NP_366_^+^, D^b^PA_244_^+^, D^b^PB1_703_^+^, and D^b^PB1‐F2_62_^+^ CD8^+^ T cells derived from each organ as indicated. Data represent 4–17 mice in each group from 12 experiments. To distinguish lung‐resident T cells, anti‐CD8α was injected i.v. 3 min prior to harvest, and cells were stained with anti‐CD8β. Data represent five mice in each group from two experiments. NT = Not tested (*P <* *0.05, **P <* *0.01, ***P <* *0.001 comparing CD8^+^ T cells specific for each peptide, at each time point, Kruskal–Wallis test).

To characterize the primary, endogenous CD8^+^ T‐cell response to IAV infection, we infected C57BL/6 mice intranasally with a nonlethal H3N2 virus, HKx31. The tissue distribution profiles of CD8^+^ T cells specific for the D^b^NP_366_, D^b^PA_224_, K^b^PB1_703_, and D^b^PB1‐F2_62_ epitopes were determined at several time points following infection by flow cytometry. Pilot studies were conducted to determine the initial arrival of IAV‐specific CD8^+^ T cells in each organ. IAV‐specific CD8^+^ T cells were first detected 4 days after infection in the MLN, but not in other tissues, with a more defined population appearing in the MLN 1 day later (Figure 1). Six days after infection, all epitope‐specific cells were detectable in the BAL, lung, liver, spleen, bone marrow, and blood (Figure 1). In general, there was a steady increase in the number of antigen‐specific CD8^+^ T cells in all sites until 8 days after infection when some populations began to decline. The lowest numbers of IAV‐specific CD8^+^ T cells were found in the bone marrow, while the highest numbers were in the lung, followed by spleen. Anderson *et al*. demonstrated that despite perfusion, the majority of CD8^+^ T cells derived from the lung were confined to the pulmonary vasculature, rather than the lung parenchyma.^20^ Therefore, to distinguish lung‐resident, IAV‐specific CD8^+^ T cells from blood‐borne cells, we performed *in vivo* labeling via intravenous injection of anti‐CD8α antibody 3 min prior to sacrificing the mice. Recovered cell suspensions were stained with anti‐CD8β antibody to distinguish blood derived (CD8α^+^CD8β^+^) cells from lung‐resident cells (CD8α^−^CD8β^+^).^20^ This analysis revealed that approximately two‐thirds of the total CD8^+^ T cells recovered from the lung were in the parenchyma. However, the overall kinetics of expansion of IAV‐specific CD8^+^ T cells in the lung was unaltered by removing blood‐borne CD8^+^ T cells from analysis (Figure 1, lung—resident and blood). Overall, the lung parenchyma and spleen harbored the most IAV‐specific CD8^+^ T cells 7 days after infection, but the spleen dominated the response 3 days later.

Analysis of the different TCR specificities showed that the D^b^PB1‐F2_62_‐specific CD8^+^ T cells were the least common throughout the response (Figure 1a). The K^b^PB1_703_‐specific CD8^+^ T cells dominated the initial response in several tissues and remained co‐dominant throughout the course of the infection in the BAL and lung, but not at other sites. While the D^b^PA_22_‐specific population was the first to reach the peak of its expansion, the D^b^NP_366_‐specific T cells were “slow starters”, and were outnumbered by all other epitope‐specific T cells in most organs early in the response, but became more prominent later in the response. Intriguingly, the kinetics of the response to each epitope does not correlate well with predicted estimates of the level or rate of antigen processing and presentation. It is estimated that NP_366_ is presented the most rapidly after infection followed by NS2_114_ and PB1‐F2_62,_ then PB1_703,_ and finally PA_224_.^21^ Thus, other factors such as the precursor frequency, the cell type presenting the antigen, the duration of antigen presentation, and the location of antigen presentation likely contribute to the immunodominance hierarchies.

To assess the relative proportion of IAV‐specific CD8^+^ T cells at each site, we compared the frequency of tetramer‐binding cells within the CD8^+^ T cell compartment of each organ. The lung, BAL, and liver had the highest frequency of IAV‐specific cells within the CD8^+^ population (Figure 1b). In contrast, IAV‐specific cells accounted for only a small proportion of the CD8^+^ T cells in the MLN, spleen, bone marrow, and blood. This is likely due to the large numbers of naïve CD8^+^ T cells in the lymphoid organs. Together, these data show that the hierarchy of CD8^+^ T cells specific for each epitope varies at different time points and between various tissues, suggesting that the magnitude of each response is not solely determined by the frequency of naïve precursors prior to infection, which is consistent with other reports.

### Recently primed IAV‐specific CD8^+^ T cells express a CD25^+^CD43^+^ phenotype

**Figure 2:**
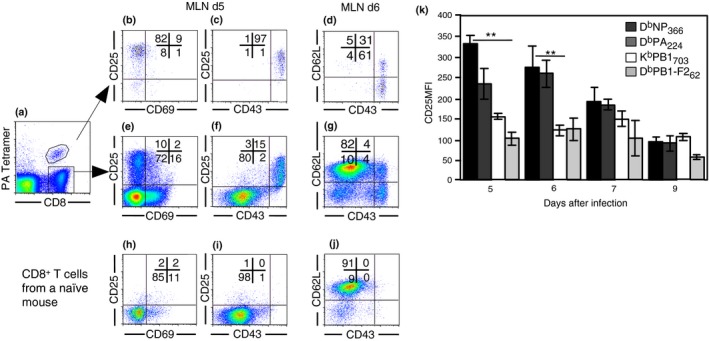
Recently primed IAV‐specific CD8^+^ T cells express a CD25^+^CD43^+^ phenotype. Lymphocytes were isolated from the MLN 5 and 6 days after intranasal (i.n.) HKx31 infection and analyzed for **(a)** CD8 expression and D^b^PA_224_ tetramer binding. Expression of **(b)** CD25 *versus* CD69, **(c)** CD25 *versus* CD43, and **(d)** CD62L *versus* CD43 on electronically gated CD8^+^ D^b^PA_224_^+^ cells. **(e)** CD25 *versus* CD69, **(f)** CD25 *versus* CD43, **(g)** CD62L *versus* CD43 on gated D^b^PA_224_^−^ CD8^+^ cells. **(h)** CD25 *versus* CD69, **(i)** CD25 *versus* CD43, **(j)** CD62L *versus* CD43 on CD8^+^ T cells from naïve mice. Data represent 4–17 mice per group from 11 independent experiments. **(k)** Each bar represents the mean ± s.e.m. for the mean fluorescence intensity of CD25 expression on gated D^b^NP_366_, D^b^PA_366_, D^b^PB1_366_, and D^b^PB1‐F2 tetramer^+^ CD8^+^ cells. Data represent 3–5 mice per group, from four experiments. (**P <* *0.01, comparing CD8^+^ T cells binding each tetramer, at each time point, Kruskal–Wallis test).

To identify a set of activation markers associated with IAV‐specific CD8^+^ T cells during the initial phase of the immune response, we assessed the expression of CD25, the active form of CD43, CD69, and CD62L on D^b^PA_224_‐specific CD8^+^ T cells in the MLN, 5 and 6 days following HKx31 infection. The majority of the D^b^PA_224_ tetramer‐binding CD8^+^ T cells in the MLN were CD25^+^CD69^−^ and CD25^+^CD43^+^ (Figure 2a–c). The expression of CD25 coincided with CD43 expression on both the tetramer‐positive (Figure 2c) and D^b^PA_224_ tetramer‐negative cells (Figure 2f). Five days following HKx31 infection, 16% of the total CD8^+^ T‐cell population had a CD25^+^CD43^+^ phenotype and this population was absent in naïve mice that were not infected with influenza (Figure 2i), suggesting that this is a distinct population of CD8^+^ T cells responding to infection. Six days after infection, a proportion of D^b^PA_224_^+^ ‐specific CD8^+^ T cells in the MLN were CD43^+^CD62L^+^. As effector CD8^+^ T cells downregulate CD62L prior to exiting the LN, this suggests that CD43 is upregulated before CD62L downregulation (Figure 2d). Overall, the CD25^+^CD43^+^ phenotype was the most homogenous population within the subset of IAV‐specific CD8^+^ T cells, highlighting it as a potential indicator of early responding virus‐specific CD8^+^ T cells.

The duration of TCR stimulation during priming dictates the level of CD25 expression.^22^ We therefore compared CD25 expression for each epitope‐specific subset. CD25 expression was higher on the D^b^NP_366_‐ and D^b^PA_224_‐specific T cells compared to K^b^PB1_703_ and D^b^PB1‐F2_62_ ‐specific T cells 5 and 6 days after infection, indicating that within the CD25^+^CD43^+^ subset, the CD25 expression level varies between the different epitopes (Figure 2k). However, the majority of all tetramer‐binding cells were CD25^+^CD43^+^ during the initials days following infection. The increased CD25 MFI on the D^b^NP_366_‐and D^b^PA_224_‐specific T cells may be a consequence of their higher avidity TCRs or increased exposure to antigen, which allows the cells to expand more than the K^b^PB1_703_ and D^b^PB1‐F2_62_ ‐specific T cells, and eventually dominate the response.

### Priming in the MLN leads to expansion of the largest population of IAV‐specific CD8^+^ T cells

**Figure 3:**
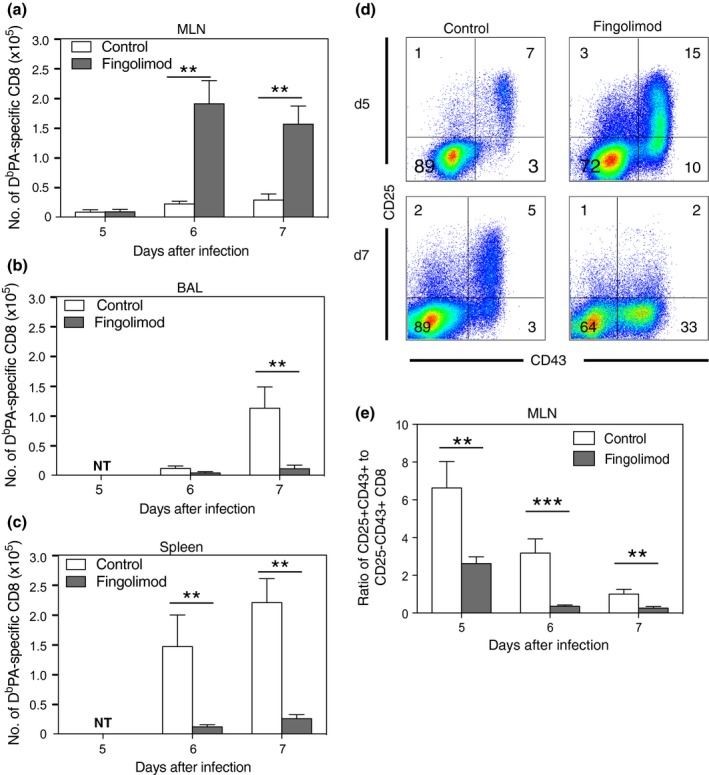
CD25^+^CD43^+^ CD8^+^ T cells progress to the CD25^−^CD43^+^ phenotype. Mice received 1 mg kg^−1^ of Fingolimod or PBS i.p. daily, beginning 52 h after i.n. HKx31 infection. Cells were recovered 5, 6 or 7 days after infection and analyzed for D^b^PA_366_ tetramer binding and CD25, CD43, and CD8α expression. **(a)** Results show the mean ± s.e.m. number of D^b^PA_244_^+^ CD8^+^ T cells from MLN, **(b)** BAL or **(c)** spleen. (*n* = 6 or 7 mice per group from 1 experiment) *P <* *0.05, **P <* *0.01 (Mann–Whitney *U‐*test). **(d)** Representative density plots show CD25 *versus* CD43 expression on gated MLN CD8^+^ T cells. **(e)** Results show the mean ± s.e.m. ratio of CD25^+^CD43^+^ to CD25^−^CD43^−^ CD8^+^ T cells.

CD25 is transiently expressed immediately following activation, prior to cell division,^23^, ^24^ while the active form of CD43 is maintained on effector cells until progression to a memory phenotype.^25^ Therefore, it is likely that during the course of the infection, the CD25^+^CD43^+^ CD8^+^ T cells progresses to the CD25^−^CD43^+^ phenotype; alternatively, these may be two distinct cell populations. To test whether CD25^+^CD43^+^ cells in the MLN become CD25^−^CD43^+^ over time, we blocked the egress of T cells from the MLN to enable us to monitor the same population of cells without cells exiting the lymph node. Fingolimod, an SIP receptor agonist^26^ was injected 52 h after intranasal HKx31 infection and then every 24 h until harvest, as previously described.^27^ The initial lag between HKx31 infection and the first Fingolimod injection allowed sufficient time for the migration of APCs from infected tissue to the lymphoid tissue. Cells were harvested from the MLN, BAL, and spleen 5, 6, and 7 days after infection. Fingolimod treatment significantly increased the numbers of D^b^PA_224_‐specific CD8^+^ T cells in the MLN, while decreasing the numbers in the BAL and spleen, indicating that egress from the MLN was blocked (Figure 3a–c). The fact that antigen‐specific CD8^+^ T cells failed to accumulate in substantial numbers at other sites when exit from the MLN was blocked, suggests that the majority of the antigen‐specific CD8^+^ T cells in the other organs are derived from the MLN. While priming in the spleen following IAV infection may contribute to the CD8^+^ T‐cell response as reported by others,^6^ our data indicate that priming outside of the MLN makes a minor contribution to the overall response. Furthermore, blocking T‐cell egress from the MLN resulted in an accumulation of CD25^−^CD43^+^ CD8^+^ T cells compared to CD25^+^CD43^+^ CD8^+^ T cells, suggesting that the cells that downregulate CD25 typically exit the MLN (Figure 3d, e). The fact that the proportion of CD25^−^CD43^+^ CD8^+^ T cells increases while the proportion of CD25^+^CD43^+^ CD8^+^ T cells decreases when entry and exit of cells into the MLN is blocked, suggests that the CD25^+^CD43^+^ cells downregulate CD25 to become CD25^−^CD43^+^, which is coincident with exit from the MLN.

### Dissemination of IAV‐specific CD8^+^ T cells

**Figure 4:**
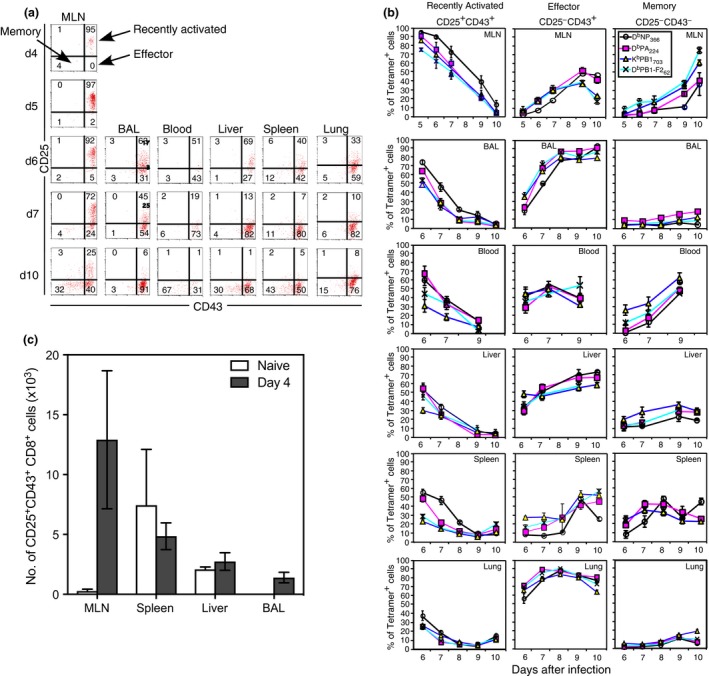
Progression of CD25 and CD43 expression by IAV‐specific CD8^+^ T cells. Lymphocytes were isolated after i.n. HKx31 infection and analyzed for tetramer binding, CD8α, CD25, and CD43 expression. **(a)** Representative density plots showing CD25 *versus* CD43 expression on electronically gated CD8^+^ D^b^PA_366_‐positive T cells for each organ, at each time point. **(b)** Each line represents the mean ± s.e.m. for the percentage of recently activated, effector, or memory tetramer‐positive CD8^+^ cells in each organ. (*n* = 4–17 mice per group, from 11 experiments). **(c)** Lymphocytes were isolated from the MLN, spleen, liver and BAL from naïve mice and mice infected i.n. with HKx31 4 days prior and analyzed for CD8α, CD43 and CD25 expression. Each bar indicates the mean ± s.e.m. number of CD25^+^CD43^+^CD8^+^ T cells. (*n* = 3–5 mice per group from 1 experiment).

To track dispersion of IAV‐specific CD8^+^ T cells following IAV infection, we assessed CD25 and CD43 expression on tetramer‐binding cells to identify where the most recently activated CD25^+^CD43^+^ T cells were located and when they progressed to an effector (CD25^−^CD43^+^) and memory (CD25^−^CD43^−^) phenotype (Figure 4a).^25^ The IAV‐specific CD8^+^ T cells were first detected in the MLN at Day 4 after infection and had a predominantly CD25^+^CD43^+^ phenotype. Six days after infection, the highest percentages of recently primed, CD25^+^CD43^+^ tetramer‐positive CD8^+^ T cells were found in the MLN (62–90%), followed by the BAL (50–75%) and blood (30–70%) (Figure 4b). Of the MLN populations, this activated phenotype was highest and maintained the longest on the D^b^NP_366_‐specific subset, relative to the other IAV‐specific CD8^+^ T cells. This high CD25 expression on cells in the MLN, again suggests that the MLN is the site of initial T‐cell priming following IAV infection. To further address this, we assessed the number of CD25^+^CD43^+^CD8^+^ T cells in the spleen, liver, and BAL at Day 4 after infection (Figure 4c). The number of cells present in these organs at this time was negligible compared to those obtained at later time points as depicted in Figure 1. Furthermore, the number of CD25^+^CD43^+^ CD8^+^ T cells present in the spleen, liver, and BAL was similar between naïve mice and infected mice, suggesting that in these organs, there is either a very low level of CD25^+^CD43^+^ CD8^+^ T cells or background staining contributes to the numbers. In contrast, there was an increase in CD25^+^CD43^+^ CD8^+^ T cells in the MLN 4 days after infection compared to naïve mice, consistent with responding cells first appearing in the MLN. Together, these data suggest that the CD8^+^ T cells were primed in the MLN and subsequently migrated to the BAL and blood, followed by the liver and spleen, and finally the lung.

### The recently primed CD25^+^CD43^+^ phenotype reveals a population of epitope‐specific CD8^+^ T cells that does not bind tetramers

**Figure 5:**
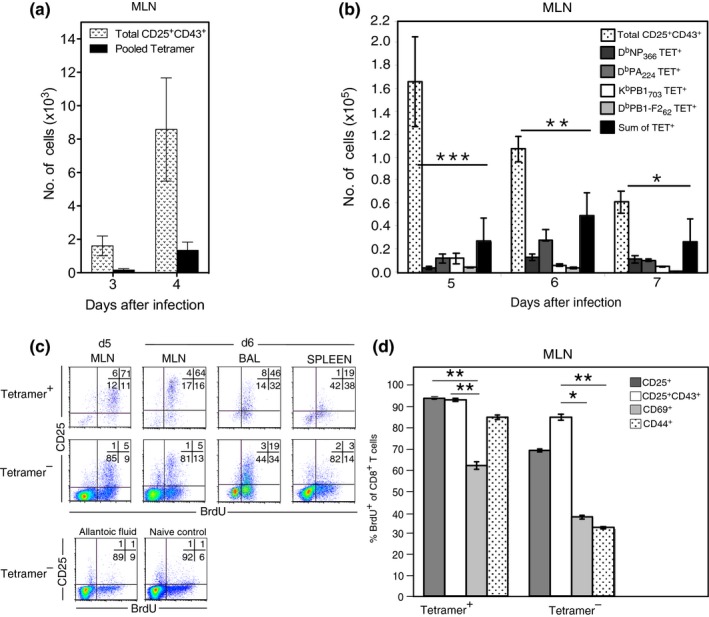
A proportion of CD25^+^CD43^+^ IAV‐specific CD8^+^ T cells do not bind tetramers. Lymphocytes were isolated from the MLN after i.n. HKx31 infection and analyzed for tetramer binding, CD8α, CD43 and CD25 expression. Each bar indicates the mean ± s.e.m. number of CD8^+^ T cells isolated on the indicated days following infection comparing **(a)** the number of CD25^+^CD43^+^CD8^+^ T cells and pooled tetramer positive CD8^+^ T cells at each time point (*n* = 4 mice per group from 1 experiment) *P <* *0.05, **P <* *0.01 (Mann–Whitney *U‐*test) or **(b)** the number of CD25^+^CD43^+^CD8^+^ T cells and sum of the tetramer positive CD8^+^ T cells at each time point (*n* = 7–14 mice per group from 9 experiments) *P <* *0.05, **P <* *0.001 (Mann–Whitney *U*‐test). **(c)** Mice were infected i.n with HKx31 and fed BrdU in the drinking water continuously. Lymphocytes were isolated from the MLN 5 and 6 days after infection and analyzed for tetramer binding, CD8α and CD25 expression, and BrdU incorporation. Representative density plots show CD25 expression *versus* BrdU incorporation on pooled tetramer‐positive and tetramer‐negative CD8^+^ T cells. Cells derived from mice injected with allantoic fluid or naïve mice served as controls. **(d)** Recovered lymphocytes were also stained with antibodies specific for CD25, CD43, CD69, and CD44. Each bar indicates the mean ± s.e.m. percentage of activated CD8^+^ T cells with a BrdU^+^ phenotype. (*n* = 4 mice per group from two experiments). *P <* *0.05, **P <* *0.01, ***P <* *0.001 comparing percentage BrdU^+^ for each set of activation markers (Kruskal–Wallis test).

To determine whether there were influenza‐specific CD8^+^ T cells that were not detected by tetramer binding, we next compared the number of CD25^+^CD43^+^ CD8^+^ T cells with the number of CD8^+^ T cells that bound the four tetramers. Few tetramer‐positive CD8^+^ T cells were observed in the MLN 3 or 4 days after infection (Figure 5a). By Day 5, tetramer‐positive CD8^+^ T cells were detectable in the MLN **(**Figure 5b). However, the number of CD25^+^CD43^+^ CD8^+^ T cells in the MLN greatly exceeded the sum of D^b^NP_366_, D^b^PA_224_, K^b^PB1_703_, and D^b^PB1‐F2_62_ tetramer‐binding CD8^+^ T cells (Figure 5a, b). This discrepancy between tetramer binding and numbers of recently activated CD25^+^CD43^+^ CD8^+^ T cells was particularly pronounced early in the response. These data suggest that either tetramer binding does not identify the entire IAV‐specific population of CD8^+^ T cells, or alternatively, only a subset of the CD25^+^CD43^+^ CD8^+^ T cells is IAV specific.

To examine how many cells within the CD25^+^CD43^+^ CD8^+^ population were IAV‐specific, we assessed the proportion of CD25^+^CD43^+^, CD25^+^, CD69^+^, and CD44^+^ CD8^+^ T cells that proliferated following IAV infection via BrdU incorporation. The majority of CD25^+^ CD8^+^ T cells in the MLN, BAL, and spleen of infected mice were BrdU^+^, indicating that these cells proliferated in response to viral infection (Figure 5c). This was true for both the subset that bound the four tetramers and the tetramer‐negative subset. As the CD25^+^ tetramer‐negative T cells proliferated following IAV infection, it is likely that they are IAV‐specific since antigen encounter is required for T cell proliferation. This was even more striking in the MLN CD25^+^CD43^+^ population of tetramer‐negative cells where 85% of the tetramer‐negative cells was BrdU^+^ (Figure 5d). In contrast, less than 40% of the CD44^hi^ or CD69^hi^ cells that did not bind tetramers divided during the course of infection. Together, these data suggest that the CD25^+^CD43^+^ phenotype identifies recently activated IAV‐specific CD8^+^ T cells.

### Recognition of viral epitopes drives expansion of CD25^+^CD43^+^ CD8^+^ T cells

**Figure 6:**
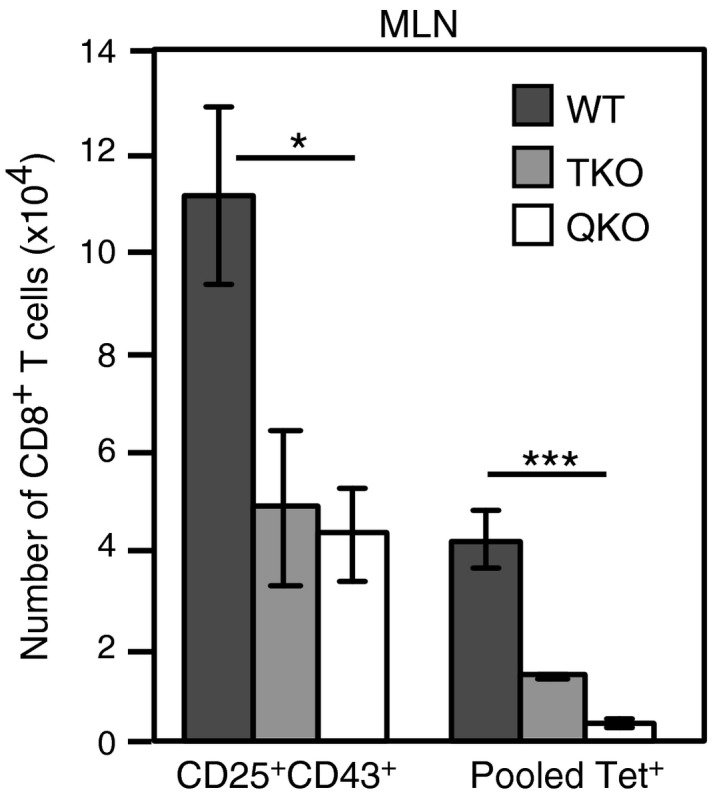
Expansion of CD25^+^CD43^+^CD8^+^ T cells correlates with dominant CD8 epitope levels. Lymphocytes isolated from the MLN, 6 days after i.n. infection with wildtype (WT), Triple knockout (TKO) (lacking the D^b^NP_366_, D^b^PA_244_, and D^b^PB1‐F2_62_ epitopes) or Quintuple knockout (QKO) (lacking the D^b^NP_366_, D^b^PA_244_, D^b^PB1_703,_ and D^b^PB1‐F2_62,_ and, K^b^NS2_114_ epitopes) virus were analyzed for tetramer binding, along with CD8α, CD25, and CD43 expression. Each bar represents the mean ± s.e.m. for the number of CD8^+^ T cells with a CD25^+^CD43^+^ phenotype, or that bind either the pooled D^b^NP_366_, D^b^PA_244_, D^b^PB1_703_, and D^b^PB1‐F2_62_ tetramers. (*n* = 6–7 mice per group from two experiments). *P <* *0.05, ***P <* *0.001 (Kruskal–Wallis test).

To further investigate whether the CD25^+^CD43^+^ CD8^+^ T cells are IAV‐specific, mice were infected with IAVs deficient in the dominant CD8 epitopes. These viruses were generated by reverse genetics that either encoded all of the HKx31 CD8^+^ T‐cell epitopes [wild‐type virus (WT)], lacked the D^b^NP_366_, D^b^PA_224, and_ D^b^PB1‐F2_62_ epitopes [Triple knockout (TKO)] or alternatively, lacked the D^b^NP_366_, D^b^PA_224,_ D^b^PB1_703_, D^b^PB1‐F2_62_, and, K^b^NS2_114_ epitopes [Quintuple knockout (QKO)].^14^ The TKO virus grows as efficiently as the WT virus *in vivo*; however, growth of the QKO virus is reduced, although it induces a productive infection.^14^ Because of the lack of the dominant CD8 epitopes, the magnitude of the overall CD8 response is reduced following infection with the TKO and QKO viruses compared to the wild‐type virus. As expected, the number of tetramer‐positive CD8^+^ T cells was significantly lower in mice infected with the TKO or QKO viruses compared to the wild‐type virus (Figure 6). Interestingly, the number of CD25^+^CD43^+^CD8^+^ T cells was also significantly reduced following infection with the TKO and QKO viruses compared to the WT virus, showing a correlation between the magnitude of CD25^+^CD43^+^CD8^+^ population and the number of CD8 epitopes presented (Figure 6). Although the CD8^+^ T cells specific for the five dominant determinants account for the majority of the responding CD8^+^ T‐cell population, recognition of other viral genes may contribute to the recently activated CD25^+^CD43^+^ CD8^+^ T‐cell population that is observed in the TKO and QKO viruses. Importantly, if the CD25^+^CD43^+^ CD8^+^ T cells were a result of nonspecific inflammation, we would expect to see similar numbers of CD25^+^CD43^+^ CD8^+^ T cells following infection with the wild type or TKO and QKO viruses. However, the results show a clear association between the number of viral epitopes presented and the numbers of CD25^+^CD43^+^CD8^+^ T cells generated, further supporting the notion that the CD25^+^CD43^+^CD8^+^ T cells are IAV specific.

### The majority of recently primed, IAV‐specific CD8^+^ T cells do not bind to tetramers

**Figure 7:**
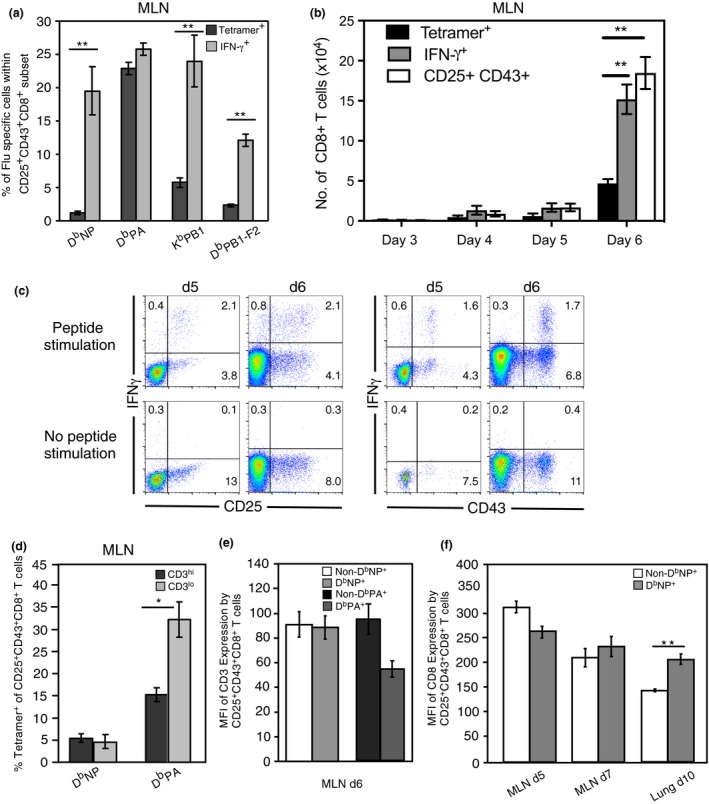
The majority of the early arising MLN CD25^+^CD43^+^ D^b^NP_366_‐specific CD8^+^ T cells evade detection with tetramer. **(a)** Lymphocytes isolated from the MLN 6 days after i.n. HKx31 infection were analyzed for the mean ± s.e.m. percentage of CD25^+^CD43^+^CD8^+^ T cells that bound tetramer or, in a separate assay, synthesized IFN‐γ following *in vitro* peptide stimulation. **P <* *0.01, comparing tetramer binding and cytokine synthesis (Mann–Whitney *U*‐test). **(b)** Each bar represents the mean ± s.e.m. number of CD8^+^ T cells with a CD25^+^CD43^+^ phenotype, cells that bound tetramer, or in a separate assay, cells that produced IFN‐γ^+^ when stimulated with each of the peptides. Data represent five groups of pooled MLN from three experiments. *P <* *0.05, comparing tetramer binding and cytokine synthesis (Kruskal–Wallis test). For Days 3–5, data represent the tetramer staining with pooled tetramers or pooled stimulation peptides. In the Day 6 time point, the data represent combined totals for each tetramer and peptide stimulation. **(c)** Representative density plots showing CD25 and CD43 expression *versus* IFN‐γ synthesis on electronically gated CD8^+^ T cells on either Day 5 or Day 6 after infection following a 5 h *in vitro* stimulation with and without peptides. **(d)** The mean ± s.e.m. percentage of CD25^+^CD43^+^CD8^+^ T cells that bound tetramer is given for gated CD3^hi^ and CD3^lo^ cells. Data represent 5 mice per group, from two experiments. (*P <* *0.05, comparing tetramer binding by each population of electronically gated cells, Kruskal–Wallis test). **(e)** Each bar represents the mean ± s.e.m. for the mean fluorescence intensity of CD3 expression on gated CD25^+^CD43^+^CD8^+^ T cells that were negative or positive for the D^b^NP_366_ tetramer, or negative or positive for the_,_ D^b^PA_366_ tetramer. Data represent 5 mice per group, from one experiment. **(f)** Each bar represents the mean ± s.e.m. for the mean fluorescence intensity of CD8 expression on gated CD25^+^CD43^+^CD8^+^ T cells, derived from the MLN and lung, that were negative or positive for the D^b^NP_366_ tetramer. Data represent 4–7 mice per group, from four experiments.

To further assess the degree of antigen‐specificity associated with the CD25^+^CD43^+^ phenotype, we compared the proportion of CD25^+^CD43^+^ cells that bound tetramer with the proportion that secreted IFN‐γ following a short *in vitro* restimulation with cognate peptide. Six days after infection, cells from the MLN were harvested and stimulated *in vitro* with the D^b^NP_366_, D^b^PA_224,_ D^b^PB1_703_, and D^b^PB1‐F2_62_ peptides, in the presence of Brefeldin, for 5 h. Only antigen‐specific T cells that have recently been activated will produce IFN‐γ in this time frame. We found the percentage of CD25^+^CD43^+^ CD8^+^ T cells that were IFN‐γ^+^ was significantly greater than the percentage that bound tetramer for all but the D^b^PA_224_‐specific response (Figure 7a). These data indicate that tetramer binding grossly underestimates the frequency of IAV‐specific CD8^+^ T cells during the early immune response to IAV. Furthermore, comparing frequency of IFN‐γ‐secretors to tetramer‐binders for each epitope showed that the D^b^PA_224_ tetramer gave the most accurate measure of epitope‐specific CD8^+^ T‐cell frequencies, and the D^b^NP_366_ tetramer was the least accurate. This may reflect the higher avidity of D^b^PA_224_‐specific CD8^+^ T cells for the corresponding peptide.^13^ While the number of CD8^+^ T cells that bound the pooled tetramers was significantly lower than the total number of CD25^+^CD43^+^ CD8^+^ T cells, the number of IFN‐γ secreting cells approximated the number of CD25^+^CD43^+^ CD8^+^ T cells (Figure 7b). Prior to Day 5 after infection, the number of IAV‐specific cells was low, consistent with their initial arrival in the MLN. These data indicate that the CD25^+^CD43^+^ phenotype more accurately reflects the number of IAV‐specific CD8^+^ T cells than tetramer staining at this early time point. Analysis of the IFN‐γ secreting cells showed that at Day 5 after infection they had a predominantly CD25^+^CD43^+^ phenotype. By Day 6, the IFN‐γ secreting cells showed some downregulation of CD25; however, CD43 was maintained (Figure 7c). This is consistent with the fact that IAV‐specific CD8^+^ T cells initially have a CD25^+^CD43^+^ phenotype, which gives rise to a CD25^−^CD43^+^ phenotype.

To determine whether CD25^+^CD43^+^CD8^+^ cells fail to bind the tetramer because of TCR downregulation, we assessed tetramer binding and CD3 expression on the CD25^+^CD43^+^CD8^+^ T cells. If cells failed to bind the tetramer because of TCR downregulation, the percentage of tetramer binding cells in the CD3^lo^ population should be reduced, compared to the CD3^hi^ population. For the D^b^NP_366_ tetramer, a similar proportion of CD3^hi^ and CD3^lo^ cells bound the tetramer, suggesting that a reduction in CD3 does not impact binding of the D^b^NP_366_ tetramer (Figure 7d). Surprisingly, for the D^b^PA_224_ tetramer, a higher proportion of the CD3^lo^ cells bound tetramer than the CD3^hi^ cells, indicating that a reduced level of CD3 does not impede tetramer binding. We also compared the mean fluorescence intensity of CD3 and CD8 expression between CD25^+^CD43^+^CD8^+^ T cells in the MLN that bound tetramer and those that did not. The levels of CD3 expression on CD25^+^CD43^+^CD8^+^ T cells were not significantly different between the D^b^NP_366_ tetramer and non‐D^b^NP_366_ tetramer binding cells (Figure 7e), suggesting that failure to bind the D^b^NP_366_ tetramer in the early responding MLN cells is not due to TCR downregulation. Similarly, CD8 expression levels were not significantly different between the D^b^NP_366_ tetramer‐ and non‐D^b^NP_366_ tetramer‐binding cells (Figure 7f), indicating that tetramer binding is not restricted by CD8 downregulation in the MLN. Furthermore, CD8 expression levels were comparable between the recently activated CD25^+^CD43^+^ subset and the resting CD25^−^CD43^−^CD8^+^ T cells (data not shown), demonstrating that there is not a significant level of CD8 downregulation following activation at this time point. In contrast, activated CD25^+^CD43^+^ CD8^+^ T cells isolated from the lung 10 days after infection showed a significant reduction in CD8 expression levels (Figure 7f). Thus, cells recovered from the infection site may fail to bind the tetramer as a result of CD8 downregulation.

### VβTCR sequence conservation indicates tetramer‐negative cells are antigen‐specific

To further examine the specificity of CD25^+^CD43^+^ CD8^+^ T cells, we analyzed the sequences of the variable region of the TCRβ chain in the D^b^NP_366_ tetramer‐positive CD25^+^CD43^+^ cells and the pooled tetramer‐negative CD25^+^CD43^+^ populations. If the CD25^+^CD43^+^ CD8^+^ T cells are IAV specific, we would expect to see similar TCR sequences in both populations. Alternatively, if the CD25^+^CD43^+^ CD8^+^ T cells are not IAV specific, we would not expect an overlap in the TCR sequences between the two populations. It was previously shown that approximately 30–60% of the D^b^NP_366_‐specific T cells are Vβ8.3^+^ with canonical CDR3β clonotype usage.^28^ Therefore, we focused the analysis on Vβ8.3^+^ CD25^+^CD43^+^ D^b^NP_366_ tetramer‐positive and Vβ8.3^+^ CD25^+^CD43^+^ pooled tetramer‐negative populations. We sorted single cells from the MLN 6 days after HKx31 infection and found that the dominant CDR3β sequence in each mouse was shared between the D^b^NP_366_ tetramer‐binding and tetramer‐negative, CD25^+^CD43^+^ CD8^+^ T cells (Table 1). This finding is extremely unlikely to occur by chance and is indicative of a shared TCR specificity. In mouse 1, two sequences dominated the response in the D^b^NP_366_ tetramer‐binding population and these were also dominating sequences in the pooled tetramer ‐negative population. In mouse 2 and mouse 3, the same “public” NP‐specific clonotype, commonly observed in D^b^NP_366_‐specific Vβ8.3^+^ repertoires,^29^ was heavily dominant to the same degree in both tetramer‐positive and negative populations. The consistency of sequence sharing among all three animals further supports our hypothesis that the tetramer‐negative CD25^+^CD43^+^ CD8^+^ T‐cell population contains IAV‐specific cells that are unable to bind tetramer, and further indicates that the CD25^+^CD43^+^ phenotype is an early indicator of virus‐specific cells.

**Table 1:** CDR3β TCR analysis of CD25^+^CD43^+^Vβ8.3 D^b^NP_366_ Tet^+^ and Tet^−^ MLN populations

CDR3β Sequence	Mouse 1		Mouse 2		Mouse 3	
	Tetramer+ (24)	Tetramer− (17)	Tetramer+ (15)	Tetramer− (22)	Tetramer+ (15)	Tetramer− (27)
SGGANTGQL			91%	87%	89%	73%
SDAGTTEV	35%	25%				
SGGSNTGQL	29%	13%				

Cells were harvested from the MLN on d6 after infection. CD25^+^CD43^+^CD8^+^Vβ8.3^+^ cells were electronically gated and D^b^NP_366_‐positive and D^b^NP_366_‐negative populations were single cell sorted for CDR3β analysis. The total number of sequences obtained for each sorted population is listed in parentheses and the frequency of the dominant sequence expressed as a percentage of the total response. (n = 3 from 1 experiment). The Tetramer‐positive cells were 12.4% ± 1.8% Vβ8.3^+^, while the percentage of Vβ8.3^+^ in the Tetramer‐negative CD25^+^CD43^+^ CD8^+^ T‐cell population was 6.3% ± 1.0%.

## Discussion

Generating long‐lived virus‐specific CD8^+^ T‐cell memory is critical to the success of a vaccine strategy eliciting T cell‐mediated immunity. Over the last decade, early priming events have been found to be critical determinants in generating these populations. More recently, it has become clear, that the magnitude of the CD8^+^ T‐cell response is also impacted by precursor frequency of a given TCR specificity, the affinity of that TCR for its ligand, the capacity of a cell to proliferate, and competition with T cells of other specificities.^9^, ^18^ Determining the impact of each of these factors on expansion of endogenous TCRs is difficult because of the low number of cells specific for each epitope.

Here, we identified a population of CD8^+^ T cells with a CD25^+^CD43^+^ phenotype that were predominantly IAV‐specific based on several criteria. First, the CD25^+^CD43^+^ cells that did not bind tetramers made IFN‐γ following a short *in vitro* restimulation with cognate peptide. Second, TCR sequences were conserved between the CD25^+^CD43^+^ cells that bound tetramer and the CD25^+^CD43^+^ cells that did not bind tetramer, suggesting that these populations share common TCR specificities. Third, infection with viruses that lack the dominant CD8 epitopes generated fewer tetramer‐positive and fewer CD25^+^CD43^+^ T cells, showing a correlation between the number of CD25^+^CD43^+^ and tetramer‐positive T cells. Finally, the fact that the CD25^+^CD43^+^ T cells proliferated after infection suggests that these cells are IAV‐specific as T cells that are not specific for IAV should not proliferate in the absence of TCR stimulation. Together, these data support the contention that the CD25^+^CD43^+^ phenotype can be used as a marker to track recently activated IAV‐specific CD8^+^ T cells.

Using the CD25^+^CD43^+^ phenotypic biomarker, we analyzed the activation and dispersion of IAV‐specific CD8^+^ T cells and found that tetramer staining underestimated the number of IAV‐specific T cells during the early phases of the response. Based on the CD25^+^CD43^+^ phenotype, approximately 7–15% of the total CD8^+^ T‐cell population in the MLN, 5 days after primary HKx31 infection, was IAV‐specific, which is four to eight times greater than the estimate from tetramer staining. Our study therefore highlights the limitation of using tetramers to quantify early antigen‐specific responses, and offers an alternative phenotype to track.

The inability of the IAV‐specific CD8^+^ T cells to bind tetramer early in the response could be due to a number of factors. As naïve T cells can effectively bind tetramers, events associated with activation may hinder tetramer binding.^13^ Indeed, several groups showed lack of tetramer binding on antigen‐specific T‐cell populations following activation.^30^, ^31^, ^32^, ^33^ One study reported that antigen‐specific T cells fail to bind tetramer following stimulation because of transient CD8 downregulation and altered CD8 glycosylation.^30^, ^31^ However, we did not observe decreased CD8 expression on the CD25^+^CD43^+^ T cells that did not bind tetramer. While tetramer binding can be influenced by CD8 staining protocols,^34^ in our experiments, tetramer binding was unaltered when anti‐CD8 antibody was incubated with or following tetramer binding (data not shown), suggesting that tetramer binding was not limited by competitive or cooperative binding. Another study tracking CD8^+^ T cells with a transgenic TCR demonstrated that IAV‐specific T cells failed to bind tetramer for up to 5 days after IAV infection, although the TCR was only downregulated for 1 day.^35^ In this study, the lack of tetramer binding was associated with altered organization of the TCR, such that the cells that bound tetramer displayed tight clustering of the TCR, while the tetramer‐negative cells had a more diffuse TCR organization. Moreover, these authors also demonstrated that disruption of the lipid rafts impacts tetramer binding.^32^ Thus, it is possible that the clustering of the TCR is required for T cells with a lower affinity for antigen to bind tetramer, and that T‐cell activation impacts the ability to cluster the TCR. The decreased TCR clustering could be due to the presence of high levels of antigen, differential glycosylation of surface proteins including CD8, or altered interaction with cytoskeletal proteins. Our data are consistent with this model, as we did not see a difference in overall TCR levels on CD25^+^CD43^+^ T cells that bound tetramer *versus* the CD25^+^CD43^+^ T cells that did not bind tetramer.

Consistent with the previous reports, we found that tetramer‐binding efficiency increased after 5 days following infection, with a greater proportion of IAV‐specific CD8^+^ T cells binding tetramer at 7 days after infection compared to the earlier time points. Selective expansion of higher affinity T cells may contribute to this increase in tetramer‐binding efficiency over time. While individual TCRs do not undergo affinity maturation, the responding population as a whole demonstrates selective expansion of CD8^+^ T cells over time.^3^

Our data indicate that the CD25^+^CD43^+^CD8^+^ T cells that did not bind tetramer were still functional as shown by IFN‐γ synthesis following *in vitro* stimulation. This is in contrast to previous reports, which used TCR transgenic T cells and *in vitro* cell lines, and found decreased tetramer binding concomitant with diminished IFN‐γ synthesis during the early immune response.^33^ We previously reported that tetramer staining parallels IFN‐γ secretion at later time points in the MLN, lung, liver, and spleen.^36^ Interestingly, in this study, we observed significant downregulation of CD8 by nontetramer binding CD25^+^CD43^+^CD8^+^ T cells recovered from the lung 10 days after infection, suggesting CD8 downregulation may be more prominent at later time points at the infection site. Xiao *et al*.^31^ demonstrated that CD8 downregulation in their system was due to type 1 interferon signaling. Thus, it is possible that the CD8 downregulation we observed in the lung is due to higher levels of type 1 interferon compared to the other organs.

The D^b^NP_366_ and D^b^PA_224_‐specific CD8^+^ T cells bound tetramers more efficiently than the less prevalent IAV‐specific CD8^+^ T‐cell populations did. Higher avidity TCRs may give the D^b^NP_366_ and D^b^PA_224_‐specific CD8^+^ T cells a proliferative advantage during clonal expansion and also make tetramer binding more efficient. A recent study of CD4^+^ T cells showed that T cells of intermediate avidity made the greatest contribution to pathogen clearance as they were less subjected to control of antigen reactivity via TCR downregulation.^37^ Similarly, the recall response of the higher avidity D^b^PA_224_‐specific CD8^+^ T cells may be suppressed in an effort to circumvent excessive damage to the host. Interestingly, CD25 expression was highest and maintained the longest on the D^b^NP_366_‐specific CD8^+^ T cells, followed by the D^b^PA_224_‐specific population. As stronger priming signals delivered to a naïve T cell increases its expansion during a recall response and elevates CD25 levels,^10^, ^22^ stronger priming signals delivered to D^b^NP_366_ ‐specific CD8^+^ T cells may contribute to their immunodominance in the recall response. Additionally, the elevated levels of CD25 may make the D^b^NP_366_‐specific CD8^+^ T cells more competitive for IL‐2, enabling them to overcome their low precursor frequency to eventually dominate the primary response. Indeed, a study reported that maintenance of CD25 expression on D^b^NP_366_‐specific CD8^+^ T cells contributed to their robust secondary proliferation and dominance in the secondary response.^38^

Priming in the MLN was identified as the main site for generating IAV‐specific CD8^+^ T cells. Using progression of CD25 and CD43 expression to follow migration, the IAV‐specific CD8^+^ T cells appeared next in the blood and BAL, then spleen and liver, followed by the lung. Similarly, TCR transgenic HSV‐1‐specific T cells had undergone multiple rounds of division by the time they trafficked to the spleen from the draining LN, with little evidence of antigenic stimulation in the spleen.^39^ Our findings are also consistent with mathematical modeling, which predicts that a greater proportion of activated CD8^+^ T cells migrate from the spleen to the lung, rather than from the MLN directly to the lung.^40^

Expression of CD43 is maintained on effector cells, correlating with increased cytolytic capacity, and downregulated on memory cells.^25^ In addition, CD43^lo^ memory cells are 2–5 fold more efficient than CD43^hi^ cells at mediating the recall response.^41^ Interestingly, we found that CD43 was gradually downregulated on IAV‐specific T cells in all organs except the BAL, lung, and liver. Accordingly, the BAL, lung, and liver are rich in effector cells, while IAV‐specific CD8^+^ T cells in the MLN and spleen give rise to the memory cells. Expression of CD43 has been associated with increased apoptosis^42^; therefore, the CD8^+^ T cells in the BAL and lung would be the most susceptible to apoptosis, as previously reported.^36^ This suggests that the lung and BAL are enriched for virus‐specific effector CD8^+^ T cells that are more susceptible to death, while, the spleen serves as a significant source of memory CD8^+^ T cells. However, some reports indicate that memory cells in the spleen cannot provide long‐term protection, therefore increasing the reliance on lung resident memory cells.^43^, ^44^ Interestingly, CD43 expression was significantly greater on the splenic D^b^NP_366_‐specific CD8^+^ T cells compared to other epitope‐specific CD8^+^ T cells in the spleen. Similar to the expression of CD25, this may contribute to the immunodominance of this population in the recall response. Thus, preventing downregulation of CD25 and CD43 may enhance trafficking of CD8^+^ T cells to the lung and BAL, and therefore prolong the generation of effector cells and increase the efficacy of a CD8^+^ T cell‐mediated IAV vaccine.

The use of surface markers to identify virus‐specific cells is not a new concept. Indeed, 7 days following infection with vaccinia virus, greater than 90% of the virus‐specific cells identified with DimerX staining expressed a GzmB^hi^CD62^Lo^ phenotype.^45^ Similarly, integrin CD11a expression and CD8α downregulation were identified as stable markers for tracking TCR‐transgenic cells specific for LCMV.^46^ However, CD11a expression is not always distinct and the extent of CD8α downregulation is proportional to the strength of antigen recognition, and varies with the nature of stimulation. Unlike the stable and ubiquitous marker CD11a, in our study, the transient nature of CD25 expression allowed us to monitor progression of the acute CD8^+^ T cells response following priming, while CD43 downregulation allowed us to distinguish between effector and memory CD8^+^ T cells.

Similar to use of other surfaces markers as surrogates for tetramers, the CD25^+^CD43^+^ biomarker clearly has limitations, including the fact that it is limited to identifying recently activated, virus‐specific cells. Nonetheless, given that not all recently activated T cells will bind tetramer, the CD25^+^CD43^+^ biomarker is useful for identifying antigen‐specific T cells early after activation. The CD25^+^CD43^+^ phenotype may also be valuable for assessing recently primed cells for responses to unknown viral antigens or for studying responses for which tetramers are not available. This biomarker may also have application for sorting or enriching for virus‐specific cells without activation through tetramer binding or the use of TCR transgenics. Importantly, the CD25^+^CD43^+^ phenotype identifies an inclusive population of early responding population of cells, which may provide insights for TCR repertoire selection and expansion. CD8^+^ T cell‐based vaccines remain an unrealized goal, efforts to understand and utilize the early events of an immune response, in particular the trafficking and maintenance of memory CD8^+^ T cells in the lung, may increase the likelihood of a cell‐mediated vaccine coming to fruition.

## Methods

### Animals, viruses and infection

Female 8–10‐week old C57BL/6J mice were purchased from the Jackson Laboratory and held under specific pathogen‐free conditions at St. Jude Children's Research Hospital. Animal studies met the approval of the St. Jude Children's Research Hospital Animal Ethics Committee. Mice were anesthetized by i.p. injection with Avertin (2,2,2‐tribromethanol; prepared in house) prior to intranasal infection. The primary response to IAV was analyzed following intranasal infection of naïve mice with 3 × 10^6^ egg 50% infective dose units of the A/HKx31 (HKx31, H3N2) IAV. Recombinant viruses, WT HKx31 and mutant HKx31(–NP –PA –F2) (TKO), and HKx31(–NP –PA –F2 –PB1 –NS2) (QKO) were generated, using the eight‐plasmid reverse genetics system described previously for the mutation of the D^b^NP_366–374_ and D^b^PA_224–233_ epitopes.^47^, ^48^ In the TKO viruses, the WT D^b^PB1‐F2_62–70_ epitope (LSLRNPILV was changed to LSLRQPILV.^49^ In the QKO virus, the WT K^b^PB1_703–711_ epitope (SSYRRVPGI) was changed to SSFRRVPGI, and the WT K^b^NS2_114–121_ epitope (RTFSFQLI) was changed to RTFSAQLI.

### Organ removal, lymphocyte recovery, and Intravascular staining

Mice were killed by CO_2_ asphyxiation and lymphocytes were recovered from the MLN, BAL, spleen, liver, lung, and blood as previously described.^36^ Femurs and tibias were removed, and the bone marrow was flushed out with 5–10 mL of RP10 medium (RPMI 1640 with 10% FCS). To distinguish lung resident and blood borne CD8^+^ T cells, mice were given tail vein injections of 3 μg of anti‐CD8α‐APC (clone 53–6.7 from eBioscience) and sacrificed 3 min later, followed by tissue harvest, as described.^20^

### Flow cytometric analysis and tetramer staining

Single‐cell suspensions were incubated for 60 min at room temperature with an APC‐conjugated tetramer specific for the D^b^NP_366_, D^b^PA_224_, K^b^PB1_703_, or D^b^PB1‐F2_62_ epitope. Cells were stained simultaneously with all four tetramers for “pooled tetramer” analysis. Following tetramer staining, cells were incubated for 20 min at 4°C with anti‐CD8α (53–6.7), and a combination of antibodies specific for CD43 (1B11), CD25 (PC61), CD69 (H1.2F3), and CD62L (MEL‐14)**.** Anti‐mouse CD16/CD32 mAb (2.4G2) was included to block nonspecific Fc‐receptor‐mediated binding. Flow cytometry data were acquired on an upgraded five‐color FACScan, FACScaliber, or LSRII (Becton Dickinson, Franklin Lakes, NJ, USA) and analyzed with FlowJo software (TreeStar, Ashland, OR, USA).

### Peptide stimulation and intracellular staining

All IAV peptides (D^b^NP_366–374_, D^b^PA_224–233_, K^b^PB1_703–711_, and D^b^PB1‐F2_62–70_) were synthesized at the Hartwell Center, St. Jude Children's Research Hospital. Lymphocytes were cultured for 5 h in a 96 well round bottom plate at a concentration of 5–10 × 10^5^ cells per well in 200 μL of RP10 containing 5 mg mL^−1^ brefeldin A (Epicenter) and 1 μm of peptide, or without peptide for unstimulated controls. After culture, cells were stained with mAb specific for CD8α (53–6.7), CD25 (PC61), CD43(1B11), and mouse CD16/CD32 then fixed in 1% formaldehyde in PBS for 30 min at room temperature. Following fixation, the cells were resuspended in 0.5% saponin (Sigma) for 10 min, incubated with fluorescently labeled anti‐IFN‐γ (XMG1.2) for 30 min at 4°C, followed by flow cytometric analysis.

### BrdU incorporation and detection

Naïve, HKx31‐virus infected, and allantoic fluid injected (virus control) mice were given sterile drinking water containing BrdU (Sigma Chemical Co., St Louis, MO) at 0.8 mg mL^−1^, which was changed daily and protected from light exposure. Recovered lymphoid cells were first stained for the expression of surface markers, as described above, then washed twice followed by fixing and permeabilizing with the BD Cytofix/Cytoperm reagents (BD Pharmingen, San Jose, CA, USA). The cells were washed again and incubated with 50 Kunz units of DNase (Sigma) for 10 min at 37°C to expose incorporated BrdU. After further washing in BD Perm/Wash buffer (BD Pharmingen) the cells were incubated with anti‐BrdU‐FITC in BD Perm/wash Buffer (BD Pharmingen) for 20 min at room temperature.

### Isolation of single CD8^+^ T cells, RT‐PCR and sequencing

Single‐cell suspensions were stained with tetramer and surface markers as described above. Lymphocytes were sorted with a MoFlo sorter (Cytomation, Fort Collins, CO) fitted with a Cyclone single‐cell deposition unit. Single immune CD8^+^CD25^+^CD43^+^Vβ8.3^+^D^b^NP_366_ or CD8^+^CD25^+^CD43^+^Vβ8.3^+^ D^b^NP_366_^−^, D^b^PA_224_^−^, K^b^PB1_703_^−^, and D^b^PB1‐F2_62_^−^ samples were sorted directly into a 96‐well PCR plate (Eppendorf, Hauppauge, NY, USA). Rows 11 and 12 of the 96 well plate were not sorted into and were used as negative controls. 60–80 cells were sorted per plate. After sorting, plates were stored at −80°C. cDNA synthesis was performed in 5 μL of cDNA reaction mixture 90 min at 37°C, followed by 5 min at 95°C. The Vβ8.3 cDNA was amplified by rested PCR, and the purified PCR products were sequenced.

### Fingolimod treatment

Fingolimod (FTY720, Cayman Chemical, Ann Arbor, MI, USA) was administered i.p. at 1 mg kg^−1^ (Cayman Chemical, Ann Arbor, MI, USA). Fingolimod was dissolved in DMSO then diluted 1:25 in PBS and 100 μL was injected 52 h after intranasal HKx31 infection and then every 24 h until harvest.

### Statistical analysis

Quantitative differences between two samples were compared with the Mann–Whitney *U‐*test (rank sum). A Kruskal–Wallis test was used, followed by Dunn's *post hoc* test, when three or more groups were compared.

## Conflict of Interest

The authors declare no conflict of interest.

## References

[1] La Gruta NL , Turner SJ . T cell mediated immunity to influenza: mechanisms of viral control. Trends Immunol 2014; 35: 396–402. 25043801 10.1016/j.it.2014.06.004

[2] Thomas PG , Keating R , Hulse‐Post DJ , Doherty PC . Cell‐mediated protection in influenza infection. Emerg Infect Dis 2006; 12: 48–54. 16494717 10.3201/eid1201.051237 PMC3291410

[3] Busch DH , Pamer EG . T cell affinity maturation by selective expansion during infection. J Exp Med 1999; 189: 701–710. 9989985 10.1084/jem.189.4.701 PMC2192934

[4] Cukalac T , Chadderton J , Handel A , *et al* Reproducible selection of high avidity CD8^+^ T‐cell clones following secondary acute virus infection. Proc Natl Acad Sci USA 2014; 111: 1485–1490. 24474775 10.1073/pnas.1323736111 PMC3910643

[5] Zehn D , Lee SY , Bevan MJ . Complete but curtailed T‐cell response to very low‐affinity antigen. Nature 2009; 458: 211–214. 19182777 10.1038/nature07657 PMC2735344

[6] Turner DL , Bickham KL , Farber DL , Lefrançois L . Splenic priming of virus‐specific CD8 T cells following influenza virus infection. J Virol 2013; 87: 4496–4506. 23388712 10.1128/JVI.03413-12 PMC3624369

[7] Williams MA , Bevan MJ . Effector and memory CTL differentiation. Annu Rev Immunol 2007; 25: 171–192. 17129182 10.1146/annurev.immunol.25.022106.141548

[8] Catron DM , Rusch LK , Hataye J , Itano AA , Jenkins MK . CD4^+^ T cells that enter the draining lymph nodes after antigen injection participate in the primary response and become central‐memory cells. J Exp Med 2006; 203: 1045–1054. 16567390 10.1084/jem.20051954 PMC2118291

[9] Jenkins MK , Moon JJ . The role of naive T cell precursor frequency and recruitment in dictating immune response magnitude. J Immunol Baltim Md 1950; 2012: 4135–4140. 10.4049/jimmunol.1102661 PMC3334329 22517866

[10] van Stipdonk MJB , Hardenberg G , Bijker MS , *et al* Dynamic programming of CD8^+^ T lymphocyte responses. Nat Immunol 2003; 4: 361–365. 12640451 10.1038/ni912

[11] Lawrence CW , Braciale TJ . Activation, differentiation, and migration of naive virus‐specific CD8^+^ T cells during pulmonary influenza virus infection. J Immunol Baltim Md 1950; 2004: 1209–1218. 10.4049/jimmunol.173.2.1209 15240712

[12] Marzo AL , Klonowski KD , Le Bon A , Borrow P , Tough DF , Lefrançois L . Initial T cell frequency dictates memory CD8^+^ T cell lineage commitment. Nat Immunol 2005; 6: 793–799. 16025119 10.1038/ni1227 PMC2849311

[13] Cukalac T , Chadderton J , Zeng W , *et al* The influenza virus‐specific CTL immunodominance hierarchy in mice is determined by the relative frequency of high‐avidity T cells. J Immunol Baltim Md 1950; 2014: 4061–4068. 10.4049/jimmunol.1301403 24696232

[14] Thomas PG , Brown SA , Keating R , *et al* Hidden epitopes emerge in secondary influenza virus‐specific CD8^+^ T cell responses. J Immunol Baltim Md 1950; 2007: 3091–3098. 10.4049/jimmunol.178.5.3091 17312156

[15] Obar JJ , Khanna KM , Lefrançois L . Endogenous naive CD8^+^ T cell precursor frequency regulates primary and memory responses to infection. Immunity 2008; 28: 859–869. 18499487 10.1016/j.immuni.2008.04.010 PMC2836785

[16] Barnes E , Ward SM , Kasprowicz VO , Dusheiko G , Klenerman P , Lucas M . Ultra‐sensitive class I tetramer analysis reveals previously undetectable populations of antiviral CD8^+^ T cells. Eur J Immunol 2004; 34: 1570–1577. 15162426 10.1002/eji.200424898

[17] Moon JJ , Chu HH , Pepper M , *et al* Naive CD4^+^ T cell frequency varies for different epitopes and predicts repertoire diversity and response magnitude. Immunity 2007; 27: 203–213. 17707129 10.1016/j.immuni.2007.07.007 PMC2200089

[18] Tscharke DC , Croft NP , Doherty PC , La Gruta NL . Sizing up the key determinants of the CD8(+) T cell response. Nat Rev Immunol 2015; 15: 705–716. 26449178 10.1038/nri3905

[19] La Gruta NL , Rothwell WT , Cukalac T , *et al* Primary CTL response magnitude in mice is determined by the extent of naive T cell recruitment and subsequent clonal expansion. J Clin Invest 2010; 120: 1885–1894. 20440073 10.1172/JCI41538 PMC2877949

[20] Anderson KG , Sung H , Skon CN , *et al* Cutting edge: intravascular staining redefines lung CD8 T cell responses. J Immunol Baltim Md 1950; 2012: 2702–2706. 10.4049/jimmunol.1201682 PMC3436991 22896631

[21] Luciani F , Sanders MT , Oveissi S , Pang KC , Chen W . Increasing viral dose causes a reversal in CD8^+^ T cell immunodominance during primary influenza infection due to differences in antigen presentation, T cell avidity, and precursor numbers. J Immunol Baltim Md 1950; 2013: 36–47. 10.4049/jimmunol.1200089 23233728

[22] Wong P , Pamer EG . Disparate *in vitro* and *in vivo* requirements for IL‐2 during antigen‐independent CD8 T cell expansion. J Immunol Baltim Md 1950; 2004: 2171–2176. 10.4049/jimmunol.172.4.2171 14764683

[23] D'Souza WN , Hedrick SM . Cutting edge: latecomer CD8 T cells are imprinted with a unique differentiation program. J Immunol Baltim Md 1950; 2006: 777–781. 10.4049/jimmunol.177.2.777 PMC3137433 16818730

[24] Oehen S , Brduscha‐Riem K . Differentiation of naive CTL to effector and memory CTL: correlation of effector function with phenotype and cell division. J Immunol Baltim Md 1950; 1998: 5338–5346. 9820507

[25] Harrington LE , Galvan M , Baum LG , Altman JD , Ahmed R . Differentiating between memory and effector CD8 T cells by altered expression of cell surface O‐glycans. J Exp Med 2000; 191: 1241–1246. 10748241 10.1084/jem.191.7.1241 PMC2193165

[26] Pinschewer DD , Ochsenbein AF , Odermatt B , Brinkmann V , Hengartner H , Zinkernagel RM . FTY720 immunosuppression impairs effector T cell peripheral homing without affecting induction, expansion, and memory. J Immunol Baltim Md 1950; 2000: 5761–5770. 10.4049/jimmunol.164.11.5761 10820254

[27] Eidsmo L , Stock AT , Heath WR , Bedoui S , Carbone FR . Reactive murine lymph nodes uniquely permit parenchymal access for T cells that enter via the afferent lymphatics. J Pathol 2012; 226: 806–813. 22170282 10.1002/path.3975

[28] Deckhut AM , Allan W , McMickle A , *et al* Prominent usage of V beta 8.3 T cells in the H‐2Db‐restricted response to an influenza A virus nucleoprotein epitope. J Immunol 1993; 151: 2658–2666. 7689611

[29] Kedzierska K , Thomas PG , Venturi V , *et al* Terminal deoxynucleotidyltransferase is required for the establishment of private virus‐specific CD8^+^ TCR repertoires and facilitates optimal CTL responses. J Immunol Baltim Md 1950; 2008: 2556–2562. 10.4049/jimmunol.181.4.2556 PMC2596983 18684946

[30] Kao C , Daniels MA , Jameson SC . Loss of CD8 and TCR binding to Class I MHC ligands following T cell activation. Int Immunol 2005; 17: 1607–1617. 16263755 10.1093/intimm/dxh340

[31] Xiao Z , Mescher MF , Jameson SC . Detuning CD8 T cells: down‐regulation of CD8 expression, tetramer binding, and response during CTL activation. J Exp Med 2007; 204: 2667–2677. 17954566 10.1084/jem.20062376 PMC2118473

[32] Drake DR , Braciale TJ . Cutting edge: lipid raft integrity affects the efficiency of MHC class I tetramer binding and cell surface TCR arrangement on CD8^+^ T cells. J Immunol Baltim Md 1950; 2001: 7009–7013. 10.4049/jimmunol.166.12.7009 11390443

[33] Spencer JV , Braciale TJ . Incomplete Cd8^+^ T lymphocyte differentiation as a mechanism for subdominant cytotoxic T lymphocyte responses to a viral antigen. J Exp Med 2000; 191: 1687–1698. 10811862 10.1084/jem.191.10.1687 PMC2193146

[34] Daniels MA , Jameson SC . Critical role for CD8 in T cell receptor binding and activation by peptide/major histocompatibility complex multimers. J Exp Med 2000; 191: 335–346. 10637277 10.1084/jem.191.2.335 PMC2195759

[35] Drake DR 3rd , Ream RM , Lawrence CW , Braciale TJ . Transient loss of MHC class I tetramer binding after CD8^+^ T cell activation reflects altered T cell effector function. J Immunol Baltim Md 1950; 2005: 1507–1515. 10.4049/jimmunol.175.3.1507 16034088

[36] Keating R , Yue W , Rutigliano JA , *et al* Virus‐specific CD8^+^ T cells in the liver: armed and ready to kill. J Immunol Baltim Md 1950; 2007: 2737–2745. 10.4049/jimmunol.178.5.2737 17312116

[37] Gallegos AM , Xiong H , Leiner IM , *et al* Control of T cell antigen reactivity via programmed TCR downregulation. Nat Immunol 2016; 17: 379–386. 26901151 10.1038/ni.3386 PMC4803589

[38] Ballesteros‐Tato A , León B , Graf BA , *et al* Interleukin‐2 inhibits germinal center formation by limiting T follicular helper cell differentiation. Immunity 2012; 36: 847–856. 22464171 10.1016/j.immuni.2012.02.012 PMC3361521

[39] Coles RM , Mueller SN , Heath WR , Carbone FR , Brooks AG . Progression of armed CTL from draining lymph node to spleen shortly after localized infection with herpes simplex virus 1. J Immunol Baltim Md 1950; 2002: 834–838. 10.4049/jimmunol.168.2.834 11777979

[40] Dobrovolny HM , Reddy MB , Kamal MA , Rayner CR , Beauchemin CAA . Assessing mathematical models of influenza infections using features of the immune response. PLoS ONE 2013; 8: e57088. 23468916 10.1371/journal.pone.0057088 PMC3585335

[41] Hikono H , Kohlmeier JE , Takamura S , Wittmer ST , Roberts AD , Woodland DL . Activation phenotype, rather than central‐ or effector‐memory phenotype, predicts the recall efficacy of memory CD8^+^ T cells. J Exp Med 2007; 204: 1625–1636. 17606632 10.1084/jem.20070322 PMC2118640

[42] Lee J‐B , Chang J . CD43 expression regulated by IL‐12 signaling is associated with survival of CD8 T cells. Immune Netw 2010; 10: 153–163. 21165244 10.4110/in.2010.10.5.153 PMC2993947

[43] Liang S , Mozdzanowska K , Palladino G , Gerhard W . Heterosubtypic immunity to influenza type A virus in mice. Effector mechanisms and their longevity. J Immunol 1994; 152: 1653–1661. 8120375

[44] Ely KH , Cookenham T , Roberts AD , Woodland DL . Memory T cell populations in the lung airways are maintained by continual recruitment. J Immunol Baltim Md 1950; 2006: 537–543. 10.4049/jimmunol.176.1.537 16365448

[45] Yuen TJ , Flesch IEA , Hollett NA , *et al* Analysis of A47, an immunoprevalent protein of vaccinia virus, leads to a reevaluation of the total antiviral CD8^+^ T cell response. J Virol 2010; 84: 10220–10229. 20668091 10.1128/JVI.01281-10 PMC2937773

[46] Rai D , Pham N‐LL , Harty JT , Badovinac VP . Tracking the total CD8 T cell response to infection reveals substantial discordance in magnitude and kinetics between inbred and outbred hosts. J Immunol 2009; 183: 7672–7681. 19933864 10.4049/jimmunol.0902874 PMC2808048

[47] Hoffmann E , Krauss S , Perez D , Webby R , Webster RG . Eight‐plasmid system for rapid generation of influenza virus vaccines. Vaccine 2002; 20: 3165–3170. 12163268 10.1016/s0264-410x(02)00268-2

[48] Webby RJ , Andreansky S , Stambas J , *et al* Protection and compensation in the influenza virus‐specific CD8^+^ T cell response. Proc Natl Acad Sci USA 2003; 100: 7235–7240. 12775762 10.1073/pnas.1232449100 PMC165859

[49] Chen W , Calvo PA , Malide D , *et al* A novel influenza A virus mitochondrial protein that induces cell death. Nat Med 2001; 7: 1306–1312. 11726970 10.1038/nm1201-1306

